# Monopolar Radiofrequency for Facial Hyperpigmentation Treatment: An Integrated Retrospective Clinical Trial and Ex Vivo Study

**DOI:** 10.3390/ijms27020761

**Published:** 2026-01-12

**Authors:** Yujin Baek, Ngoc Ha Nguyen, Seoyoon Ham, Wanjin Kim, Ju Hee Lee, Young In Lee

**Affiliations:** 1 Department of Dermatology, Cutaneous Biology Research Institute, Yonsei University College of Medicine, Seoul 03722, Republic of Korea; uj200076@yuhs.ac (Y.B.); ngocha7996@yuhs.ac (N.H.N.); hsy7852@yuhs.ac (S.H.); jinykim0917@yuhs.ac (W.K.); juhee@yuhs.ac (J.H.L.); 2 Department of Dermatology, University of Medicine and Pharmacy at Ho Chi Minh City, Ho Chi Minh City 17000, Vietnam; 3 Scar Laser and Plastic Surgery Center, Yonsei Cancer Hospital, Seoul 03722, Republic of Korea

**Keywords:** monopolar radiofrequency (MRF), pigmentation, skin aging, heat-shock proteins, basement membrane

## Abstract

Aging-associated facial hyperpigmentation is driven not only by enhanced melanogenesis but also by dermal senescence and deterioration of the dermal–epidermal junction. The purpose of this study was to evaluate whether monopolar radiofrequency (MRF) monotherapy can improve aging-related facial hyperpigmentation by simultaneously suppressing melanogenic signaling and restoring senescence-associated dermal alterations. We assumed that deep dermal heating induced by MRF would modulate fibroblast senescence and basement membrane integrity, thereby indirectly regulating melanocyte activity. In a retrospective review of 26 Asian women, MRF treatment significantly decreased multiple pigmentation parameters, including melanin level, hyperconcentration, and Hemi Melasma Area and Severity Index (hemi-MASI) scores, while concurrently reducing wrinkles, pores, and enhanced overall skin texture without inducing inflammation. Complementary ex vivo experiments using ultraviolet B (UVB)-irradiated human skin demonstrated that MRF markedly reduced pro-melanogenic markers (*α-MSH*, *MC1R*, *MITF*, *TYR*, *TRP1/2*), restored collagen type IV expression at the basement membrane, decreased senescence-associated genes (*p16*, *p21*), and upregulated protective heat shock proteins (*HSP70/47*). Together, these findings suggest that MRF improves aging-associated hyperpigmentation by both suppressing melanogenesis and rejuvenating the senescent dermal microenvironment. MRF may serve as an effective non-invasive treatment option for pigmentation disorders in aging skin.

## 1. Introduction

Melanin, particularly eumelanin, serves as a key photoprotective pigment in human skin, absorbing ultraviolet (UV) radiation and neutralizing reactive oxygen and nitrogen species [1,2]. While physiologic melanogenesis following UV exposure is essential for cutaneous defense, aging is often accompanied by heightened melanocyte activity and impaired regulatory mechanisms [3]. This dysregulation drives excessive eumelanin synthesis, leading to pigmentary disorders—including lentigines, melasma, post-inflammatory hyperpigmentation (PIH)—that collectively compromise skin aesthetics and quality of life [4]. In addition, senescent dermal fibroblasts exacerbate pigmentation by degrading the basement membrane, thereby facilitating melanocyte descent into the dermis and intensifying pigmentary cross-talk [5].

Given this interplay, energy-based therapies capable of restoring dermal architecture and revitalizing fibroblast function may offer meaningful therapeutic potential for pigmentary disorders. Emerging evidence further suggests that radiofrequency (RF)-based modalities may aid in improving hyperpigmentation. Specifically, in vitro studies have demonstrated that bipolar RF can suppress melanogenesis through diverse mechanisms, including tyrosinase inhibition, upregulation of heat shock proteins (HSP70/90), reduced adenosine triphosphate (ATP) production, attenuation of fibroblast senescence, enhancement of lymphangiogenesis, and promotion of melanosome autophagy in keratinocytes [5,6,7,8,9]. 

Notably, monopolar RF (MRF) has gained prominence in both Eastern and Western aesthetic markets due to its well-established ability to remodel collagen, enhance elasticity, reduce subcutaneous fat, stimulate fibroblast regeneration, and treat periorbital wrinkles [5,10,11,12,13,14]. Compared with bipolar RF, MRF offers a deeper extent of bulk heating, allowing for a more comprehensive skin rejuvenation that can target deep melanin deposition in the dermis [15]. Indeed, MRF in combination with 1% kojic acid decreased the Melasma Area and Severity Index (MASI) and the average melanin score after 6 sessions in 50 melasma patients [16]. Furthermore, the combined use of MRF with nonablative diode laser has also shown potential in treating dyspigmentation in another prospective, single-arm study [17]. Additionally, other types of RF have also been utilized to treat pigmentary issues, such as micro-plasma RF for post-burn hyperpigmentation [18], sublative fractional RF for periorbital hyperpigmentation [19], microneedle RF for senescence-induced hyperpigmentation [20], or invasive pulsed-type bipolar alternating current RF on melasma and rebound hyperpigmentation [21].

Despite these insights, clinical and mechanistic data specifically addressing the depigmenting efficacy of MRF monotherapy remain scarce. Therefore, the present study aims to comprehensively evaluate the effects of MRF on facial rejuvenation, with particular emphasis on hyperpigmentation reduction, through a retrospective analysis of prospectively collected trial data using two established MRF devices. In addition, an ex vivo investigation in UVB-irradiated human skin tissue was conducted to elucidate the molecular mechanisms underlying the depigmenting actions of MRF.

## 2. Results

### 2.1. Clinical Study on Monopolar RF’s Depigmentation Effects

Our single-center, retrospective, clinical study included 26 participants with a mean age of 57.54 ± 4.09 years old. Our cohort consisted of 3 males (11.54%) and 23 females (88.46%) participants (Table 1). 

#### 2.1.1. Primary Outcome: Pigmentation

**Figure 1:**
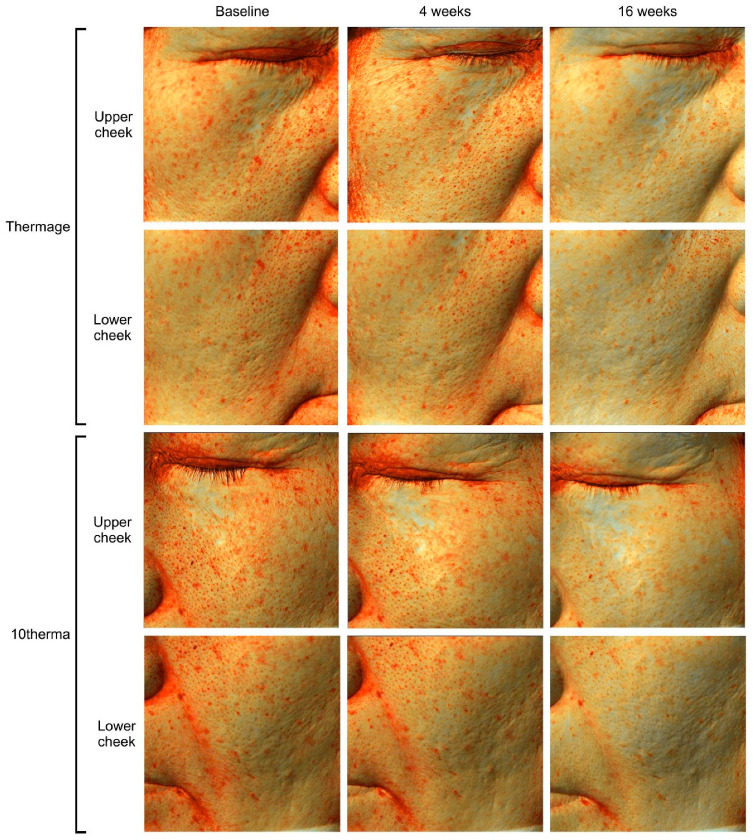
Antera 3D photographs of melanin spots on the upper and lower cheek areas at baseline, 4 weeks, and 16 weeks after treatment with Thermage or 10therma.

**Figure 2:**
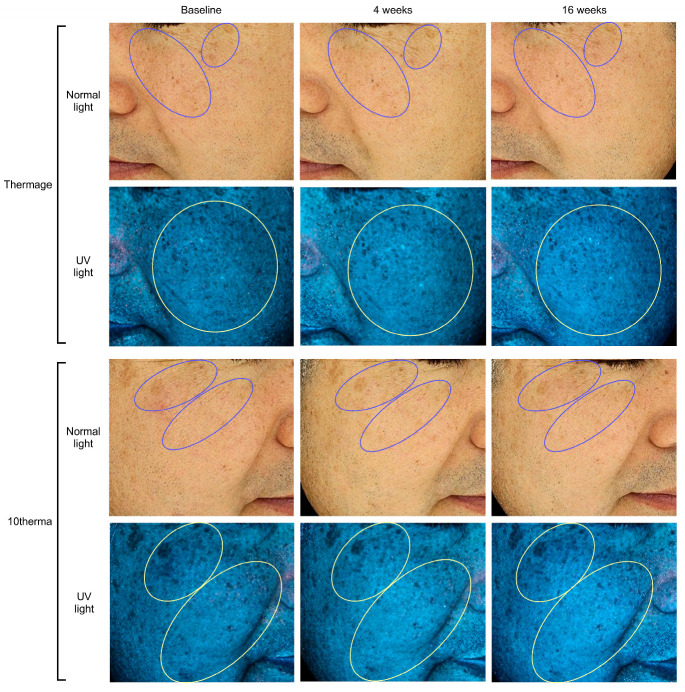
Mark-Vu photographs under normal light and UV light on the cheek areas at baseline, 4 weeks, and 16 weeks after treatment with Thermage or 10therma. The blue and yellow ellipses represent the areas with a reduction in pigmentation.

Treatment with Thermage (Solta Medical Inc., Bothell, WA, USA) or 10therma (TENTECH Inc., Seoul, Republic of Korea) both led to marked visual reductions in melanin via Antera 3D photos (Figure 1) as well as Mark-Vu images (Figure 2).

**Figure 3:**
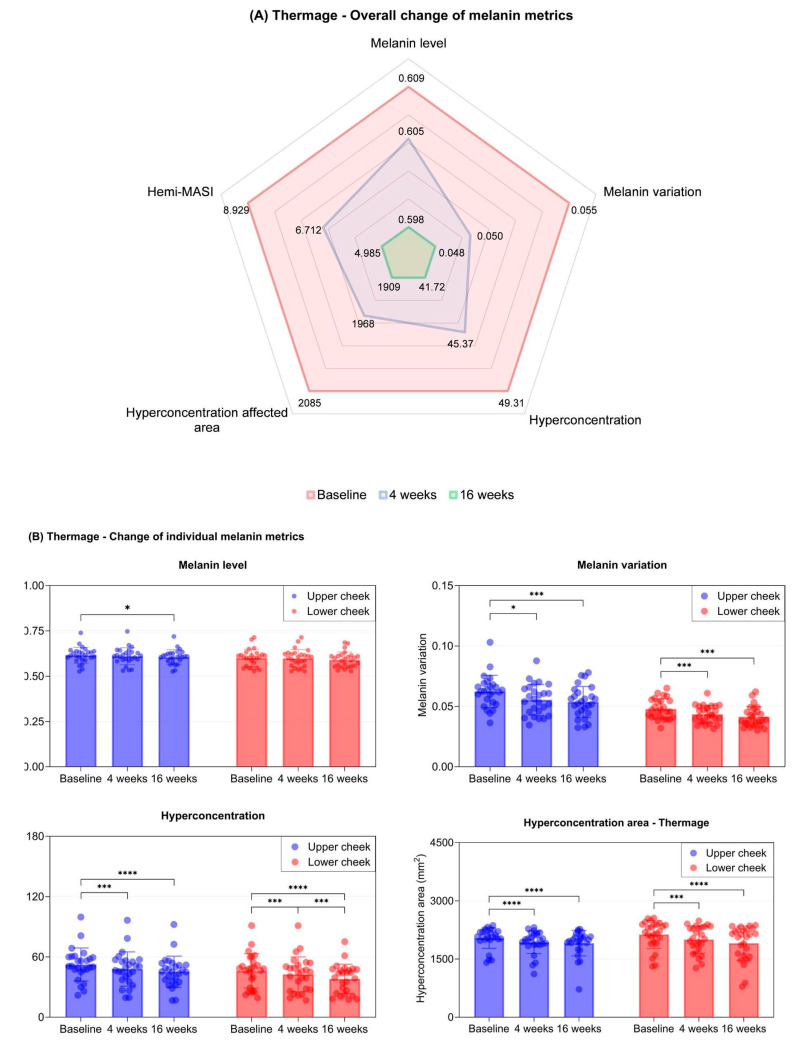
(**A**) Radar chart showing reductions in multiple melanin measurements at 4 weeks and 16 weeks after treatment with Thermage. (**B**) Bar and scatter plots showing reductions in melanin level, variation, hyperconcentration, and hyperconcentration area in the upper and lower cheek areas over 16 weeks of treatment with Thermage. Data are presented as mean ± SD, each dot represents individual values. N = 26 in each group. Asterisks indicate comparisons within group using one-way repeated measures ANOVA or the Friedman test depending on normality; * *p* < 0.05, *** *p* < 0.005, **** *p* < 0.001.

**Figure 4:**
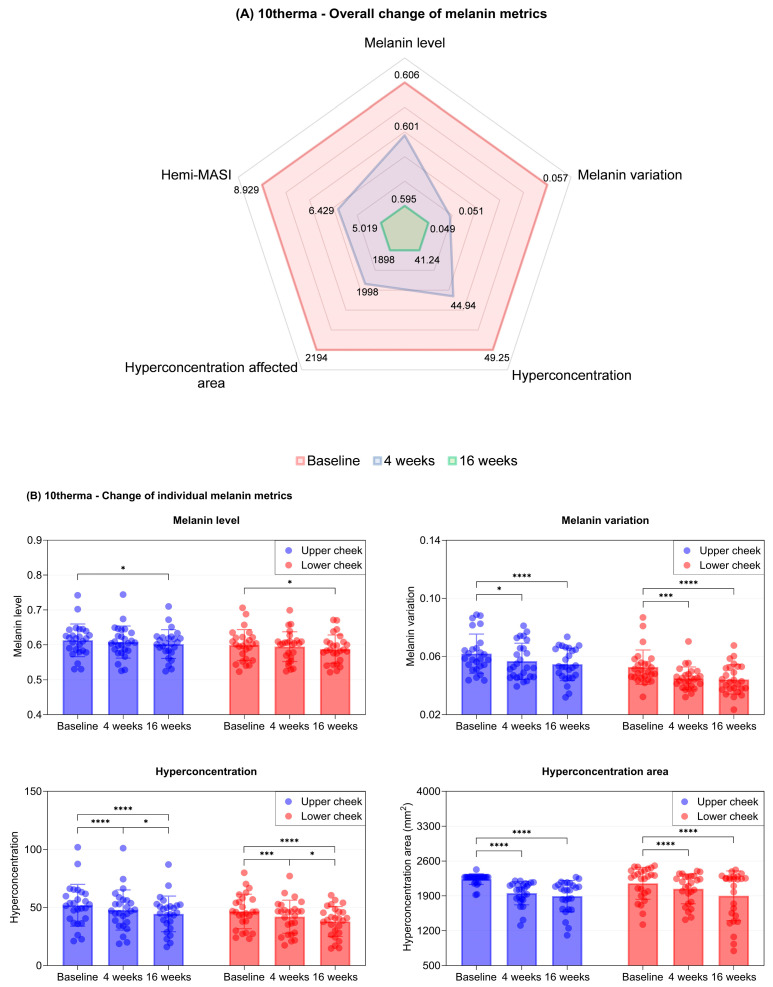
(**A**) Radar chart showing reductions in multiple melanin measurements at 4 weeks and 16 weeks after treatment with 10therma. (**B**) Bar and scatter plots showing reductions in melanin level, variation, hyperconcentration, and hyperconcentration area in the upper and lower cheek areas over 16 weeks of treatment with 10therma. Data are presented as mean ± SD, each dot represents individual values. N = 26 in each group. * Asterisks indicate comparisons within group using one-way repeated measures ANOVA or the Friedman test depending on normality; * *p* < 0.05, *** *p* < 0.005, **** *p* < 0.001.

Quantitative analysis also showed considerable reductions in wrinkle metrics in both groups (Figure 3A and Figure 4A).

Specifically, in the Thermage group, notable improvements in pigmentation were observed. At the upper cheek, all indices exhibited consistent declines, with statistical significance achieved at all time points, except for melanin level at week 4. For the lower cheek, although melanin level did not reach statistical significance, it followed a clear decreasing trend over 16 weeks. Melanin variation, hyperconcentration, and hyperconcentration area achieved significant reductions from baseline at both 4 and 16 weeks (Table 1, Figure 3B). Collectively, these results confirm that Thermage effectively reduced pigmentation throughout the study. 

In the 10therma group, we observed significant improvements across all pigmentation-related parameters. In both the upper and lower cheek regions, melanin levels demonstrated a downward trend, with statistically significant reductions from baseline at week 16. Moreover, melanin variation, hyperconcentration, and hyperconcentration area showed substantial decreases as early as week 4, with further reductions at week 16 in both facial regions (Table 1, Figure 4B). These findings indicate a marked reduction in hyperpigmentation and a more uniform melanin distribution following 10therma treatment.

**Figure 5:**
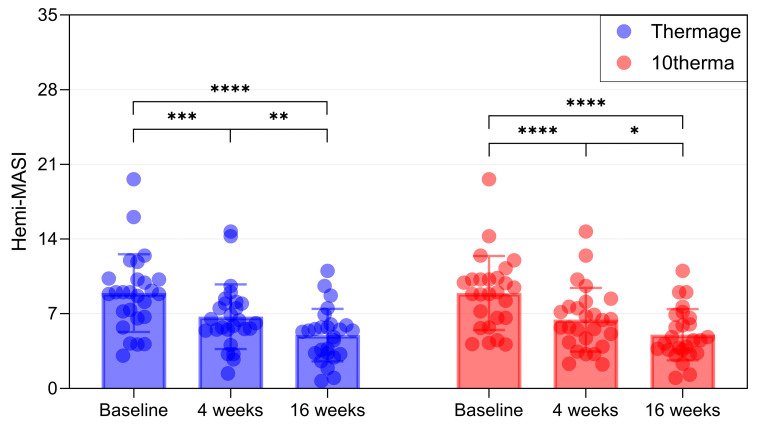
Bar and scatter plot showing reductions in hemi-MASI scores in Thermage and 10therma groups after 16 weeks. Data are presented as mean ± SD, each dot represents individual values. N = 26 in each group. * Asterisks indicate comparisons within group using one-way repeated measures ANOVA or the Friedman test depending on normality; * *p* < 0.05, ** *p* < 0.01, *** *p* < 0.005, **** *p* < 0.001.

Subjective evaluation using the hemi–Melasma Area and Severity Index (hemi-MASI) scale supported these objective findings, with both devices producing continuous and statistically significant score reductions from baseline (Table S1, Figure 5).

Regarding the relationship between the depigmentation efficacy of the treatment and age, our analysis showed no statistically significant correlation after adjusting for baseline values (Table 2).

#### 2.1.2. Secondary Outcomes: Wrinkles, Pores, Texture, Hemoglobin, and GAIS

**Figure 6:**
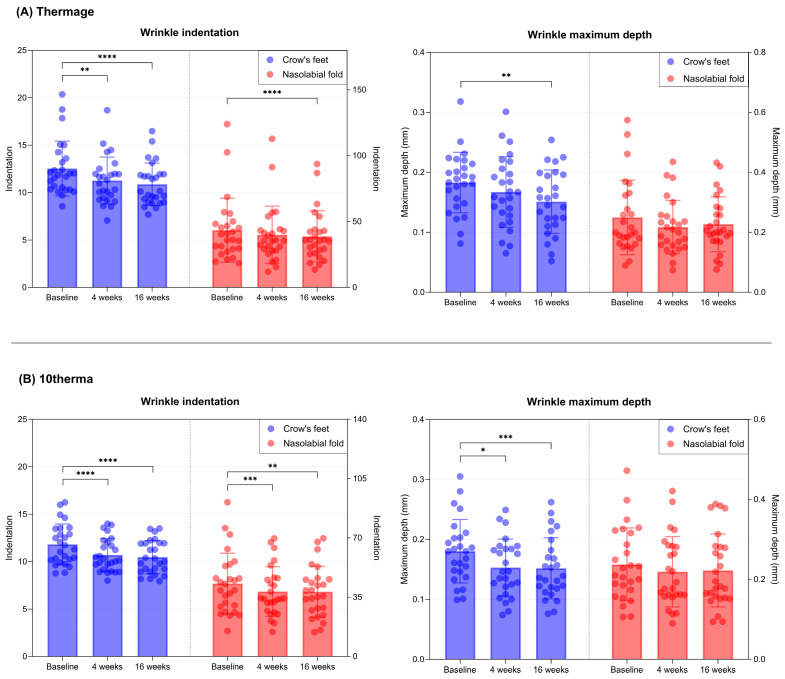
Bar and scatter plots showing reductions in wrinkle indentation index and maximum depth at the Crow’s feet and nasolabial fold over 16 weeks in the (**A**) Thermage and (**B**) 10therma groups. Data are presented as mean ± SD, each dot represents individual values. N = 26 in each group. Asterisks indicate comparisons within group using one-way repeated measures ANOVA or the Friedman test depending on normality; * *p* < 0.05, ** *p* < 0.01, *** *p* < 0.005, **** *p* < 0.001.

In the Thermage group, crow’s feet wrinkles showed significant improvement at both 4 and 16 weeks, as evidenced by decreases in both the indentation index and maximum wrinkle depth. The nasolabial fold also demonstrated a significant reduction in indentation index at each follow-up visit, although changes in maximum depth were less pronounced (Table 1, Figure 6A). 10therma treatment yielded comparable wrinkle-reducing effects, with similar improvements at the crow’s feet and nasolabial fold (Table 1, Figure 6B).

**Figure 7:**
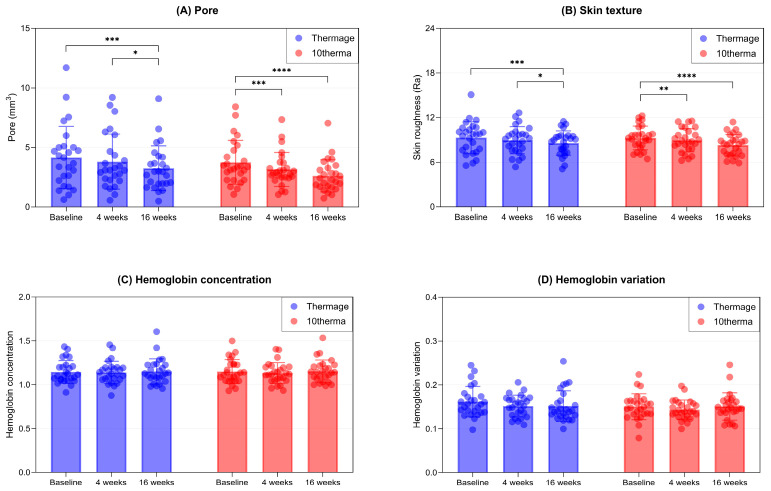
Bar and scatter plots showing (**A**) Reductions in pore volume after 16 weeks in Thermage and 10therma groups. (**B**) Improvement in skin texture/Reduction in skin roughness after 16 weeks in Thermage and 10therma groups. Changes in (**C**) hemoglobin concentration and (**D**) hemoglobin variation after 16 weeks in Thermage and 10therma groups. Data are presented as mean ± SD, each dot represents individual values. N = 26 in each group. Asterisks indicate comparisons within group using one-way repeated measures ANOVA or the Friedman test depending on normality; * *p* < 0.05, ** *p* < 0.01, *** *p* < 0.005, **** *p* < 0.001.

Additionally, both devices significantly reduced pore volume from baseline (Figure 7A), suggesting a tightening effect on skin surface structure.

Moreover, skin texture, measured by arithmetical roughness (Ra), improved consistently and significantly with both treatments (Figure 7B), indicating smoother skin surface topography.

Notably, neither device induced significant changes in hemoglobin concentration or variation (Figure 7C,D), suggesting minimal risk of vascular damage or treatment-induced inflammation.

**Figure 8:**
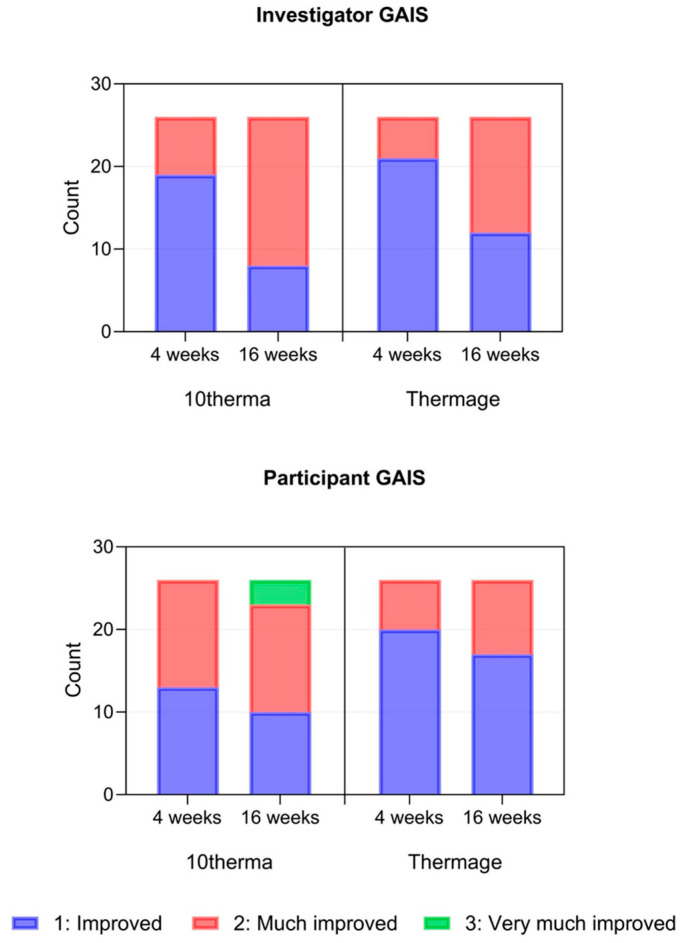
GAIS scores of the investigator and participants after 4 and 16 weeks in Thermage and 10therma groups.

Finally, the Global Aesthetic Improvement Scale (GAIS) ratings reflected high satisfaction among participants and positive assessments from the investigator for both devices at 4 and 16 weeks (Figure 8), aligning with the objective outcomes.

### 2.2. Ex Vivo Study

**Figure 9:**
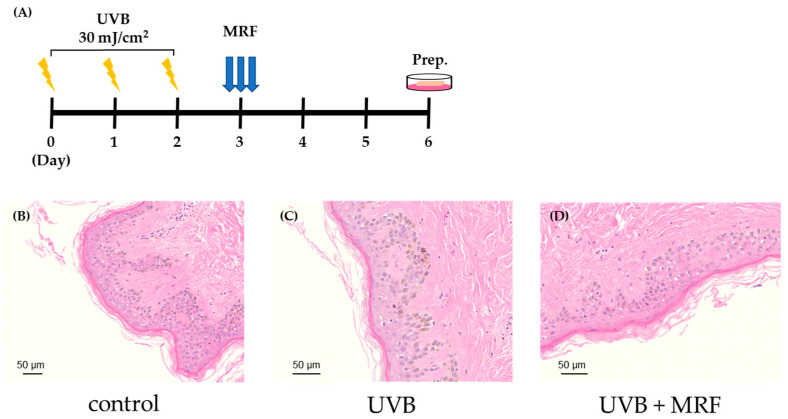
Ex vivo design and Histological analysis of skin tissues by Hematoxylin and Eosin (H&E) staining. (**A**) Experimental design and ex vivo timeline. Representative H&E-stained images of (**B**) control, (**C**) ultraviolet (UVB)-irradiated, and (**D**) UVB + monopolar radiofrequency (MRF)-treated skin tissues. UVB irradiation was associated with epidermal thickening and structural alteration, whereas UVB + MRF-treated samples showed relatively preserved epidermal and dermal morphology compared with UVB-only samples. Results from the remaining two independent experiments (a total of three biological replicates) are provided in Figure S1.

The experimental design is described in Figure 9A.

#### 2.2.1. Hematoxylin and Eosin (H&E) Staining

In the control group, the epidermis and dermis exhibited normal, well-organized structures with uniform thickness (Figure 7B). In contrast, the UVB-irradiated group showed epidermal thickening, irregular morphology, and epidermal barrier disruption, along with reduced dermal density and structural disorganization (Figure 7C). Following RF irradiation, tissue architecture was largely restored, with improved epidermal continuity and denser dermal structures (Figure 7D), indicating recovery from UVB-induced damage. 

#### 2.2.2. Fontana–Masson (FM) Staining

**Figure 10:**
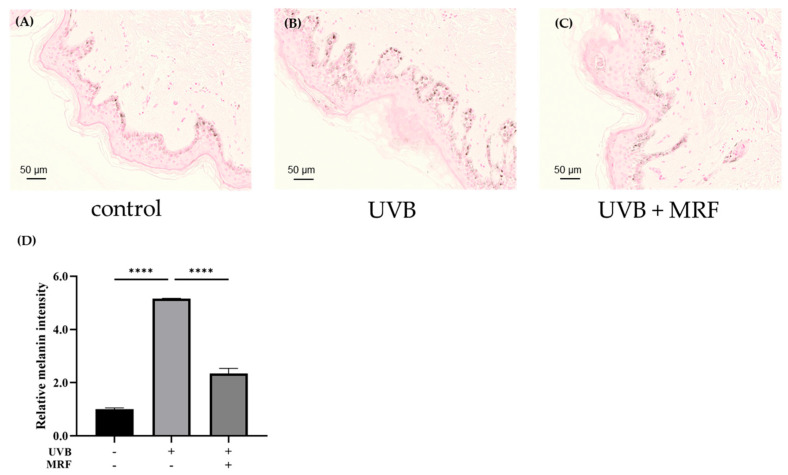
Melanin visualization by Fontana–Masson (FM) staining. Representative FM-stained images of (**A**) control, (**B**) ultraviolet (UVB)-irradiated, and (**C**) UVB + monopolar radiofrequency (MRF)-treated groups. Increased melanin deposition was observed in UVB-irradiated samples compared with controls, whereas reduced pigmentation was observed in UVB + MRF-treated samples relative to the UVB-only group. (**D**) Quantitative analysis of FM staining corresponding to panels (**A**–**C**). Data are presented with statistical significance indicated (**** *p* < 0.0001). Results from the remaining two independent experiments (a total of three biological replicates) are provided in Figure S2.

To further examine pigmentation changes, FM staining was performed. In the control group, melanin granules were evenly localized along the basal layer of the epidermis (Figure 10A). The UVB-irradiated group showed markedly increased melanin accumulation, confirming UVB-induced hyperpigmentation (Figure 10B). In contrast, the MRF-treated group exhibited a noticeable decrease in melanin content (Figure 10C), indicating that MRF irradiation effectively reduced UVB-induced pigmentation and promoted restoration of pigment homeostasis in photoaged skin.

#### 2.2.3. Immunohistochemistry (IHC) Analysis

**Figure 11:**
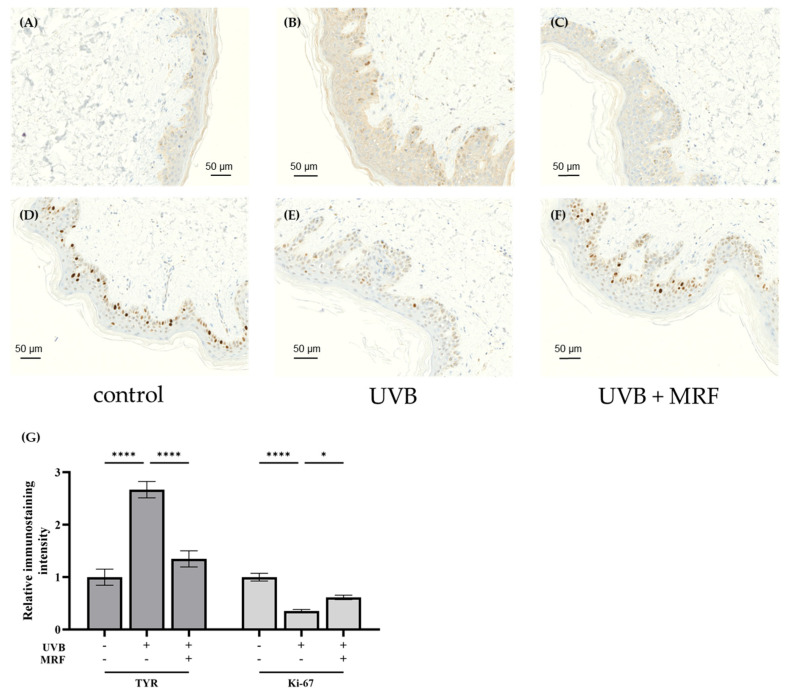
Immunohistochemical (IHC) staining of Tyrosinase (TYR) and Ki-67 in skin tissues. Representative IHC images of TYR (**A**–**C**) and Ki-67 (**D**–**F**) in control (left panel), ultraviolet (UVB)-irradiated (middle panel), and monopolar radiofrequency (MRF)-treated groups (right panel). TYR expression increased after UVB irradiation (**** *p* < 0.0001) and decreased following MRF treatment (**** *p* < 0.0001). Ki-67–positive cells decreased after UVB exposure (**** *p* < 0.0001) and increased in MRF-treated skin, indicating enhanced proliferative activity (* *p* = 0.0328). (**G**) Quantitative analysis derived from panels (**A**–**F**); statistical significance is indicated. Results from the remaining two independent experiments (a total of three biological replicates) are provided in Figure S3. TYR, Tyrosinase.

IHC staining was used to assess the effects of MRF treatment on pigmentation, dermal structure, and cellular proliferation. UVB irradiation increased tyrosinase (TYR) expression in the epidermis (Figure 11A,B), indicating enhanced melanogenesis. In contrast, MRF-treated skin showed reduced TYR expression (Figure 11C), suppressing UVB-induced melanogenesis. Ki-67 expression decreased in the UVB group (Figure 11D,E), reflecting impaired cellular renewal, but MRF-treated skin showed increased Ki-67-positive cells in the basal layer (Figure 11G), suggesting enhanced proliferation and regeneration.

#### 2.2.4. Western Blot (WB) Analysis

**Figure 12:**
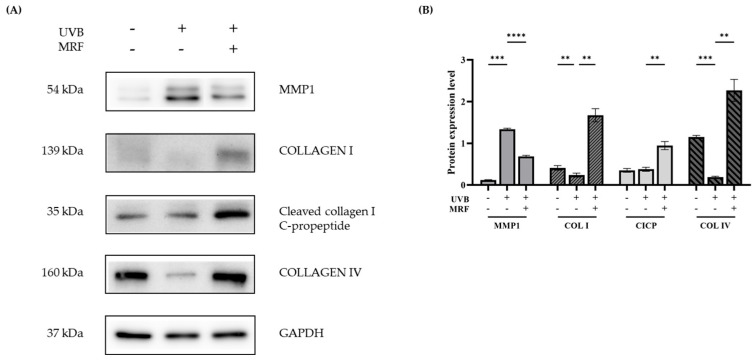
Western blot (WB) analysis. (**A**) Representative WB images of MMP1, COL I, Cleaved collagen I C-propeptide, and COL IV in control, ultraviolet (UVB)-irradiated, and monopolar radiofrequency (MRF)-treated groups. GAPDH was used as a loading control. MMP1 expression increased after UVB irradiation and decreased with MRF treatment, whereas COL I, CICP, and COL IV showed the opposite trend, indicating MRF-induced restoration of ECM proteins. (**B**) The graph shows the quantified values of (**A**), with statistical significance indicated. ** *p* < 0.01, *** *p* < 0.001, **** *p* < 0.0001. MMP-1, matrix metalloproteinase-1; COL I, type I collagen; CICP, Cleaved collagen I C-propeptide; COL IV, type IV collagen; GAPDH, glyceraldehyde 3-phosphate dehydrogenase.

WB analysis showed that metalloproteinase-1 (MMP1) expression increased after UVB irradiation and decreased following MRF treatment. In contrast, collagen type I (COL1), collagen type I c-propeptide (CICP), and collagen type IV (COL IV) levels were reduced by UVB irradiation but restored after MRF treatment (Figure 12A). These results indicate that MRF irradiation suppressed UVB-induced collagen degradation and promoted extracellular matrix recovery. These findings were consistently observed across three independent biological replicates, and all corresponding raw Western blot data are provided in Figure S4. Quantitative analysis further confirmed these effects (Figure 12B).

#### 2.2.5. Quantitative Reverse Transcription PCR (RT-qPCR) Analysis

**Figure 13:**
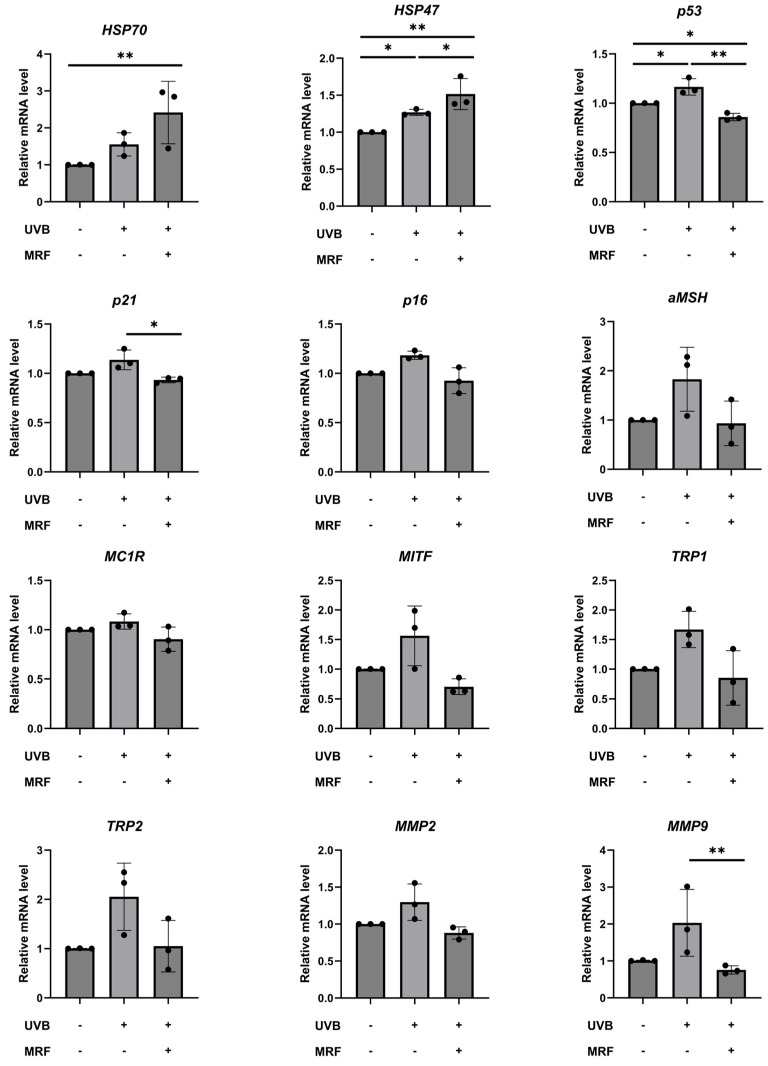
Gene expression analysis by quantitative reverse transcription PCR (RT-qPCR). Relative mRNA expression levels of *HSP70*, *HSP47*, *p53*, *p21*, *p16*, *α-MSH*, *MC1R*, *MITF*, *TRP1*, *TRP2*, *MMP2*, and *MMP9* in control, ultraviolet (UVB)-irradiated, and monopolar radiofrequency (MRF)-treated groups. UVB irradiation increased the expression of stress-, pigmentation-, and MMP-related genes, while MRF treatment reversed these changes and further enhanced heat shock–related gene expression. For each donor, technical triplicates were averaged, and statistical analyses were performed using three independent donors. Relative gene expression levels were calculated using the 2^−ΔΔCt^ method and are shown as mean ± SD (n = 3 biological replicates). Each data point corresponds to the donor-level mean derived from technical triplicates. Statistical significance was determined based on ΔCt values analyzed using a linear mixed-effects model with donor treated as a random effect. Adjusted *p*-values were applied for multiple comparisons. * *p* < 0.05, ** *p* < 0.01. HSP70, heat shock protein 70; HSP47, heat shock protein 47; p53’ tumor protein p53; p21, cyclin-dependent kinase inhibitor 1A; p16, cyclin-dependent kinase inhibitor 2A; α-MSH, alpha-melanocyte-stimulating hormone; MC1R, melanocortin 1 receptor; MITF, microphthalmia-associated transcription factor; TRP1, tyrosinase-related protein 1; TRP2, tyrosinase-related protein 2; MMP2, matrix metallopeptidase 2; MMP9, matrix metallopeptidase 9.

*Heat shock protein 70 (HSP70)*, and *heat shock protein 47 (HSP47)* levels were upregulated after UVB exposure and further increased after MRF treatment, indicating activation of heat shock–related protective responses. In contrast, the expression of *p53, p21*, and *p16* (cell cycle–related genes) and *alpha-melanocyte-stimulating hormone (α-MSH), melanocortin 1 receptor (MC1R), microphthalmia-associated transcription factor (MITF), tyrosinase-related protein 1 (TRP1),* and *tyrosinase-related protein 2 (TRP2)* (melanogenesis-related genes) was elevated by UVB irradiation but decreased following MRF treatment. Similarly, *matrix metallopeptidase 2 (MMP2)* and *matrix metallopeptidase 9 (MMP9)* expression increased after UVB exposure and was reduced after MRF irradiation (Figure 13). For each donor, RT–PCR measurements were performed in technical triplicate, which were averaged prior to analysis. Consistent gene expression patterns were observed across biological replicates obtained from three independent donors, and statistical analyses were conducted using donor-level data.

## 3. Discussion

Aging-associated pigmentation disorders remain among the most therapeutically challenging conditions in aesthetic dermatology. This retrospective study was the first to evaluate MRF for improving aging-related hyperpigmentation. The findings provide consistent clinical evidence supporting the efficacy of two commercially available MRF devices in mitigating hyperpigmentation, as indicated by significant reductions in both objective melanin indices and subjective hemi-MASI scores. The treatment demonstrated a favorable safety profile with minimal risk of vascular damage or inflammation, and achieved high satisfaction among both participants and investigators.

In comparison, resurfacing lasers—such as Q-switched 1064/532 nm and CO_2_ lasers—are well established for treating benign hypermelanosis and achieving skin rejuvenation, might yield faster visible results than RF [22]. However, their use is associated with higher risks of dyspigmentation (PIH, hypopigmentation), prolonged erythema, and scarring, particularly in darker Fitzpatrick skin types (III–VI) with increased melanocyte activity [23,24]. While both RF and laser therapies target pigmentation and photoaging, their distinct mechanisms suggest complementary roles: RF, through deep dermal heating and fibroblast modulation [25], might be advantageous for dermal and mixed-type pigmentation, whereas lasers are better suited for superficial epidermal lesions to minimize treatment-related adverse events [26]. 

Skin pigmentation is regulated by interactions among dermal fibroblasts, epidermal keratinocytes, and melanocytes, particularly under conditions of cellular senescence [27]. To investigate pigmentation-related mechanisms, we established an ex vivo model using UVB irradiation to induce cellular aging of the skin [28]. In detailed mechanism, UVB exposure activates p53-dependent α-MSH production in keratinocytes, which binds to MC1R on melanocytes, stimulating MITF and downstream melanogenic enzymes such as tyrosinase, TRP1, and TRP2 [29]. Meanwhile, HSPs function as molecular chaperones maintaining protein stability, which is upregulated under stress conditions such as UV radiation, heat, or oxidative injury [30]. In the skin, HSPs (including HSP27, HSP47, and HSP70) protect keratinocytes and fibroblasts from UV radiation by preventing protein misfolding, facilitating the degradation of irreversibly damaged proteins [30,31]. Interestingly, HSP70 also modulates cytoplasmic p53, preventing its overexpression [6]. In our study, MRF treatment mitigated UVB-induced effects on melanogenesis and upregulated HSP70. Consistent with prior findings by Kim et al. (2021) on bipolar RF [6], MRF may enhance HSP70 expression to suppress p53 activation and downregulate melanogenic signaling (α-MSH, MC1R, MITF, TRP1, TRP2), thereby directly inhibiting melanogenesis in melanocytes.

On the other hand, MRF may alleviate pigmentation via its senolytic effects. p16 and p21 are two established senescence markers commonly utilized in combination to enhance detection accuracy in quantifying UV-induced cellular senescence [32,33]. p16- or p21-positive melanocytes exhibit increased melanin content [34], while senescent fibroblasts promote melanogenesis through basement membrane degradation by MMPs and paracrine signaling with melanocytes [35,36,37]. In this study, MRF treatment reduced p16- and p21-positive cells, potentially decreasing melanin-rich aged melanocytes. The reduction in senescence indicators coupled with the increase in the activity of HSP47, a fibroblast intracellular protein that enhances collagen production, suggests rejuvenation of this cell type [38,39]. This consequently might result in the restoration of collagen IV in the dermal-epidermal junction and the lower secretion of degradative enzymes MMP2/MMP9, thereby repairing the basement membrane and inhibiting the melanocyte – fibroblast cross-talk [39,40,41]. Thus, MRF may indirectly reduce melanogenesis by remodeling the senescent dermal microenvironment, decreasing both senescent melanocytes and fibroblasts, in addition to its direct mechanism. Further studies with co-staining of aging markers and cell indicators are needed to validate these findings.

Other potential mechanisms for inhibiting melanogenesis include the downregulation of ATP production, enhancement of lymphangiogenesis, and promotion of melanosome autophagy in keratinocytes, as previously demonstrated with bipolar RF [7,8,9]. However, the validation of these effects in MRF remains to be established. 

In our study, increased expression of collagen I and its C-propeptide form correlated with reduced wrinkles and improved skin texture, as demonstrated in clinical assessments. MRF, known for its collagen-tightening properties and its rejuvenating effects on aged fibroblasts, promotes the production of collagen I in senescent skin [25]. This restoration helps reverse aging-induced wrinkles, resulting in a firmer skin barrier and smoother texture.

The depigmentation effect of MRF observed in our study aligns with those reported in previous trials. Cameli et al. (2014) combined the use of MRF and transdermally-delivered 1% kojic acid in treating 50 patients with melasma, reducing MASI scores and quantitative melanin scores [16]. An integrated MRF-diode laser modality has also shown its efficacy in alleviating facial dyspigmentation in a recent study in 2025 [17]. Other types of RF, namely microneedling RF, sublative fractional RF, micro-plasma RF, or invasive pulsed-type bipolar alternating current RF, have demonstrated their whitening effects in senescence-induced hyperpigmentation, periorbital hyperpigmentation, post-burn hyperpigmentation, and melasma, respectively [18,19,20,21]. Although many of these studies incorporated the use of another agent or device with depigmenting effects, our aligned results can corroborate the fact that MRF, as a standalone therapy, could serve as a viable and safe option to remedy pigmentation issues.

This study has several limitations. Although conducted with a randomized split-face design, its retrospective nature may have introduced selection bias, and the small sample size limits generalizability. The absence of long-term follow-up also precludes evaluation of treatment durability. Furthermore, the mechanistic findings were not derived from in vivo analyses, and additional pathways require further validation. Finally, direct real-time monitoring of tissue or culture medium temperature during RF delivery was not performed. Unlike in vivo skin, ex vivo tissue lacks perfusion-mediated heat dissipation, which may increase susceptibility to heat accumulation and non-specific thermal injury. Although no overt histological evidence of thermal necrosis was observed in H&E staining or downstream molecular analyses, the absence of real-time temperature monitoring precludes precise characterization of thermal distribution within the tissue. Future studies incorporating direct temperature measurements or infrared thermography during RF exposure would further strengthen mechanistic interpretation and help delineate biological effects from non-specific thermal damage. Moreover, future large-scale, multicenter randomized controlled studies with extended follow-up, as well as investigations directly comparing or combining monopolar RF with other energy-based devices, are warranted to confirm these findings and better define efficacy–safety profiles.

## 4. Materials and Methods

### 4.1. Clinical Study 

#### 4.1.1. Study Design and Participants

This study was conducted as a single-center retrospective analysis using data obtained from our previous split-face clinical trial that investigated the effects of MRF treatment on facial skin (manuscript in submission). The original split-face clinical study was performed at three institutions, in which one randomly assigned side of each participant’s face received 10thermage treatment, while the contralateral side underwent Thermage therapy. The purpose of the original study was to evaluate the lifting and reduction in wrinkles of two MRF devices.

In this present retrospective analysis, data exclusively from the Yonsei University cohort were reviewed to assess the overall efficacy of MRF in improving age-related hyperpigmentation (IRB number: 1-2024-0014). The dataset included clinical photographs and evaluation records of participants who had undergone MRF treatment with additional age-related pigmentation concerns.

A total of 26 participants were recruited for the study. Eligible participants were subjects aged 30–65 years who agreed to abstain from any dermatological procedures, including facial lifting, during the study and were able to adhere to the study protocol. Participants were excluded if they had keloid or hypertrophic scars, recent facial procedures (laser, phototherapy, or surgery) within six months, heat-induced disorders, or uncontrolled medical conditions. Those who used whitening agents such as hydroquinone or tranexamic acid within six months or failed to complete all follow-up visits were also excluded. All participants received a detailed explanation of the study’s objectives, procedures, and potential risks, and provided written informed consent before enrollment in accordance with the Declaration of Helsinki.

#### 4.1.2. Treatment Protocol

Before treatment, the handpiece was disinfected using ≥70% ethanol. Electrodes and return pads were inspected for defects, disinfected, and used once only; all components were disposed of after use in accordance with regulations. A dedicated conductive gel was applied to the treatment area. Treatments were generally performed without anesthesia; however, localized anesthesia using gauze was permitted based on subject skin sensitivity or upon request.

In both groups, energy was delivered at a frequency of 6.78 MHz with a maximum RF power of 400 W and an impedance operating range of 75–400 Ω. Each subject received a single treatment session for both the full face and the periocular area. In Thermage group, a 4.0 cm^2^ tip was used for facial treatment and a 0.25 cm^2^ tip for the periocular region. For facial application, energy densities were set at 15.4 J/cm^2^ (Level 2.5), 17.8 J/cm^2^ (Level 3.0), and 20.3 J/cm^2^ (Level 3.5). For the periocular area, energy densities were 36 J/cm^2^ (Level 2.0), 40 J/cm^2^ (Level 2.5), and 44 J/cm^2^ (Level 3.0). A total of 300 shots were delivered to the face and 113 shots to the periocular area, with treatment durations of 7 min and 30 s and 5 min, respectively, while maintaining the skin surface temperature between 38 °C and 42 °C.

In the 10therma group, a 5.0 cm^2^ tip was used for facial treatment and a 0.25 cm^2^ tip for the periocular area. Facial energy densities were set at 15.4 J/cm^2^ (Level 2.5), 17.8 J/cm^2^ (Level 3.0), and 20.2 J/cm^2^ (Level 3.5), while periocular energy densities were 39 J/cm^2^ (Level 2.0), 42 J/cm^2^ (Level 2.5), and 45 J/cm^2^ (Level 3.0). The number of shots, treatment times, and skin surface temperature were identical to those of the Thermage group.

#### 4.1.3. Clinical Measurements of Skin Parameters

Skin assessments were conducted at baseline (V0), at 4 weeks (V1), and at 16 weeks post-treatment (V2). At each measurement session, participants’ faces were first cleansed, then acclimatized for 30 min in a controlled environment (temperature: 20–24 °C, relative humidity: 45–55%) before undergoing evaluation.

##### Primary Outcome

We utilized the Antera 3D CS (Miravex, Dublin, Ireland), a device that captures skin images under controlled multi-directional LED light and then applies 3D surface reconstruction with spectral reflectance analysis to objectively assess pigmentation-related parameters:

- Melanin level—measures the overall concentration of melanin pigment in the skin.
- Melanin variation (uniformity)—evaluates the evenness of melanin distribution, with higher variation indicating more uneven pigmentation.
- Melanin hyperconcentration—quantifies localized areas with abnormally high melanin density.
- Melanin hyperconcentration area (mm^2^)—measures the total surface area of these highly pigmented regions.

The subjective evaluation was conducted by a certified dermatologist (N.H.N.) using the hemi-MASI scale (Table S1) based on standardized digital photographs taken via Mark Vu^TM^ (PSI Plus, Suwon-si, Gyeonggi-do, Republic of Korea), a device that captures high-resolution facial images under multiple controlled LED light sources (normal, specular, polarized, and ultraviolet).

##### Secondary Outcomes

Additional skin characteristics were quantified using the Antera 3D CS:

- Wrinkles: measured at the crow’s feet and nasolabial fold areas.
  ○ Indentation index: indicates the average depth and severity of wrinkle depressions.
  ○ Maximum wrinkle depth (mm): the deepest point of wrinkle furrows.
- Pore volume (mm^3^)—measures the total three-dimensional volume of visible skin pores, reflecting pore enlargement.
- Hemoglobin:
  ○ Concentration—quantifies skin redness, which may be related to vascularization or inflammation.
  ○ Variation—evaluates the evenness of redness distribution across the measured area.
- Texture: Arithmetical skin roughness (Ra)—calculates the average height variation of the skin surface; higher Ra values indicate rougher skin.

Subjective assessment of overall aesthetic improvement was also performed by both participants and investigators using the GAIS, which rates improvement relative to baseline appearance (Table S2).

At each visit, participants were monitored for adverse events, and all confirmed or reported cases were recorded to calculate incidence rates for the overall safety evaluation.

A summary of the clinical assessments and evaluation schedule is provided in Table S3.

### 4.2. Ex Vivo Experiments

#### 4.2.1. Ex Vivo Skin Tissue Culture and UVB/MRF Irradiation

Residual human skin specimens were obtained from healthy female donors of Korean descent, aged between 40 and 50 years, undergoing breast reconstruction, with approval from the Institutional Review Board of Severance Hospital (IRB No. 4-2025-0577). Written informed consent for the use of their tissues in research was obtained from all participants. Detailed donor demographic information, including age, sex, ethnicity, and tissue source, is provided in Table S4. 

Skin tissues were obtained from three healthy female donors. For each donor, experiments were performed in triplicate or more for each condition. Tissues were cultured in Dulbecco’s Modified Eagle Medium (DMEM) supplemented with 10% fetal bovine serum and 1% penicillin–streptomycin. Each full-thickness skin sample was trimmed to 4 × 4 cm^2^ and assigned to one of three groups: control, UVB, or UVB + MRF. Samples in the UVB and UVB + MRF groups were irradiated with UVB (UV Crosslinker, Vilber, Collégien, France) at 30 mJ/cm^2^ at 0, 24, and 48 h. After pigmentation induction, the UVB + MRF group received MRF treatment using a 5-cm^2^ tip of the 10THERMA device (level 3.5, cooling intensity 7) under standardized conditions. Immediately afterward, subcutaneous adipose tissue was carefully removed, and the tissues were placed on a DMEM-based 1% agar gel prepared by mixing complete DMEM with 5% (w/v) agar stock at a 4:1 ratio. All samples were maintained for 5 days in a humidified incubator at 37 °C with 5% CO_2_, with medium refreshed every 24 h.

#### 4.2.2. Histological Analysis 

Skin tissues were fixed in 10% neutral-buffered formalin for 24 h, embedded in paraffin, and sectioned at 4 μm to generate formalin-fixed paraffin-embedded (FFPE) slides. Stained sections were examined using a light microscope (CX33, Olympus, Tokyo, Japan) and digitally scanned with the Axio Scan.Z7 system (Carl Zeiss, Jena, Germany) at 200× magnification for image analysis.

- H&E staining, sections were processed using a commercial H&E Stain Kit (ab245880, Abcam, Cambridge, MA, USA) according to the manufacturer’s instructions to assess histological changes in the epidermis and dermis.
- FM staining, FFPE sections were subjected to standard black reduction to visualize melanin, followed by counterstaining with nuclear fast red, using the Fontana-Masson Stain Kit (ab150669, Abcam, Cambridge, MA, USA). Melanin deposition was quantified using ImageJ (version 1.46r) by calculating the percentage of melanin-positive area within the epidermis.

#### 4.2.3. Immunohistochemical (IHC)

FFPE sections were deparaffinized, rehydrated, and subjected to heat-induced epitope retrieval. For IHC, antigen retrieval was performed using Target Retrieval Solution low pH 6.0 at 95 °C for 20 min for Ki-67 and tyrosinase (TYR). Sections were treated with 3% H_2_O_2_ for 10 min, blocked with 5% normal serum for 2 h at room temperature, and incubated overnight at 4 °C with primary antibodies against Ki-67 (ab15580, Abcam, Cambridge, MA, USA; 1:500), TYR (ab738, Abcam, Cambridge, MA, USA; 1:100). After washing, slides were treated with secondary antibodies, digitally scanned at 200× magnification using the Axio Scan.Z7 system, counterstained with hematoxylin, and quantified using ImageJ. 

#### 4.2.4. Western Blot 

Tissues were homogenized using a TissueLyser II (QIAGEN, Hilden, Germany), and total protein was extracted using radioimmunoprecipitation assay buffer supplemented with a phosphatase inhibitor cocktail. Protein concentrations were determined by bicinchoninic acid assay, with absorbance measured on a microplate ELISA reader. Equal amounts of protein (20 µg) were loaded per lane, separated on 8–10% Sodium Dodecyl Sulfate Polyacrylamide Gel Electrophoresis at 80 V for 2 h, and transferred to polyvinylidene fluoride membranes. Membranes were blocked in 5% skim milk in TBST for 1 h at room temperature and incubated overnight at 4 °C with primary antibodies MMP1 (#54376, Cell Signaling Technology, Danvers, MA, USA; 1:1000), COL I (ab260043, Abcam, Cambridge, MA, USA; 1:1000), COL IV (ab6586, Abcam, Cambridge, MA, USA; 1:1000), and GAPDH (#2118, Cell Signaling Technology, Danvers, MA, USA; 1:1000). After washing, membranes were incubated with a horseradish peroxidase-linked anti-rabbit IgG secondary antibody (ab7074, Abcam, Cambridge, MA, USA; 1:1000) for 1 h at room temperature. Protein bands were detected using enhanced chemiluminescence and imaged on an Amersham ImageQuant 800 system (Cytiva, Marlborough, MA, USA). Detailed information on all antibodies used is provided in Supplementary Table S5. Original Western blot images corresponding to the quantified data are provided in Figure S4 as source data.

#### 4.2.5. Quantitative Reverse Transcription PCR (RT-qPCR)

Tissue samples were homogenized using a TissueLyser II (QIAGEN, Hilden, Germany), and total RNA was extracted with TRIzol reagent (15596018, Invitrogen, Waltham, MA, USA). RNA concentration was evaluated using a NanoDrop 2000 spectrophotometer (Thermo Fisher Scientific, Waltham, MA, USA). cDNA was synthesized from total RNA using the RNA to cDNA EcoDry Premix (Oligo dT) kit (Takara, Seoul, Republic of Korea). RT-qPCR was performed with Q Master Mix on a QuantStudio 3 Real-Time PCR System (Applied Biosystems, Waltham, MA, USA). The PCR conditions were 50 °C for 2 min, 95 °C for 10 min, followed by 40 cycles of 95 °C for 15 s, 60 °C for 1 min, and a final extension at 95 °C for 15 s, 60 °C for 1 min, and 95 °C for 15 s. Primer sequences are listed in Table S6. Relative mRNA expression was calculated using the 2^−ΔΔCt^ method, with GAPDH as the internal control. RT–qPCR analyses were performed using tissue samples obtained from three independent donors (biological replicates), and each sample was analyzed in technical triplicate. 

### 4.3. Statistical Analysis

Statistical analyses and graphing were conducted using GraphPad Prism 10.2.2, with significance set at *p* < 0.05. We expressed results as mean ± standard deviation. In the clinical trial, for normally distributed data, we applied one-way repeated measures ANOVA to evaluate statistically significant differences within each group (paired analysis), followed by Tukey’s post-hoc test for paired comparisons between each time point. For non-normally distributed data, we used the Friedman test to evaluate statistically significant differences within each group (paired analysis), with the Wilcoxon signed-rank test and Bonferroni correction for post-hoc analyses. To assess the correlation between age and the depigmentation efficacy, we used the linear regression analysis with adjustment for baseline values.

In the RT-PCR experiments, we presented relative gene expression levels, calculated using the 2^−ΔΔCt^ method, as mean ± SD (n = 3 biological replicates). We analyzed differences between groups using a linear mixed-effects model based on ΔCt values, with donor treated as a random effect. Adjusted *p*-values were applied for multiple comparisons. 

## 5. Conclusions

This integrated clinical and ex vivo study demonstrates that MRF is an effective and safe non-invasive modality for improving aging-related facial hyperpigmentation. Clinically, MRF produced significant and sustained reductions in objective melanin indices and hemi-MASI scores while simultaneously improving wrinkles, skin texture, and pore parameters, without evidence of treatment-induced inflammation or vascular injury. These findings suggest that MRF provides global skin rejuvenation benefits, including pigment reduction. Mechanistically, our ex vivo data indicate that the depigmenting effects of MRF extend beyond direct melanocyte suppression. MRF attenuated UVB-induced melanogenesis by downregulating the α-MSH–MC1R–MITF pathway and melanogenic enzymes, while also reducing cellular senescence markers and restoring dermal–epidermal junction integrity through collagen IV recovery. The concurrent upregulation of HSPs further supports a role for MRF in maintaining protein stability and enhancing dermal repair. Collectively, these results support a model in which MRF improves hyperpigmentation by rejuvenating the senescent dermal microenvironment that sustains abnormal melanocyte activity in aging skin. From a clinical perspective, these findings position MRF as a promising option for treating hyperpigmentation, particularly in patients at higher risk of post-inflammatory hyperpigmentation from laser-based therapies. However, given the retrospective design, limited sample size, and absence of long-term follow-up, larger prospective randomized studies are warranted to confirm durability, optimize treatment parameters, and define patient selection criteria. Future investigations combining in vivo mechanistic validation with long-term clinical outcomes will be essential to fully establish the role of MRF in pigmentation management strategies for aging skin.

## Figures and Tables

**Table 1:** Demographic features and clinical skin measurements of participants over 16 weeks.

	Thermage			10therma		
	Baseline	4 Weeks	16 Weeks	Baseline	4 Weeks	16 Weeks
**cAge (years)**	57.54 ± 4.09					
**Gender, n (%)**						
Male	3 (11.54)					
Female	23 (88.46)					
**Fitzpatrick skin type, n (%)**						
II	19 (73.08%)					
III	5 (19.23%)					
IV	2 (7.69%)					
**Melanin level**						
Upper cheek	0.62 ± 0.04	0.61 ± 0.05	0.61 ± 0.04	0.61 ± 0.05	0.61 ± 0.05	0.6 ± 0.04
*p*-value ^1^		0.23	**0.043**		0.124	**0.036**
*p*-value ^2^		0.578			0.347	
Lower cheek	0.6 ± 0.05	0.6 ± 0.05	0.59 ± 0.04	0.6 ± 0.04	0.59 ± 0.04	0.59 ± 0.04
*p*-value ^1^		0.7504	0.0661		0.319	**0.023**
*p*-value ^2^		0.191			0.244	
**Melanin variation**						
Upper cheek	0.06 ± 0.01	0.06 ± 0.01	0.05 ± 0.01	0.06 ± 0.01	0.06 ± 0.01	0.05 ± 0.01
*p*-value ^1^		**0.037**	**0.001**		**0.011**	**<0.001**
*p*-value ^2^		0.752			0.636	
Lower cheek	0.05 ± 0.01	0.04 ± 0.01	0.04 ± 0.01	0.05 ± 0.01	0.04 ± 0.01	0.04 ± 0.01
*p*-value ^1^		**0.003**	**0.002**		**0.001**	**<0.001**
*p*-value ^2^		0.335			0.999	
**Hyperconcentration**						
Upper cheek	52.5 ± 16.35	47.99 ± 17.05	45.25 ± 15.56	51.9 ± 18.05	47.85 ± 17.3	44.51 ± 15.32
*p*-value ^1^		**0.002**	**<0.001**		**<0.001**	**<0.001**
*p*-value ^2^		0.146			0.024	
Lower cheek	46.12 ± 17.11	42.75 ± 17.28	38.18 ± 14.55	46.61 ± 14.85	42.03 ± 14.24	37.96 ± 12.86
*p*-value ^1^		**0.004**	**<0.001**		**0.002**	**<0.001**
*p*-value ^2^		**0.002**			**0.016**	
**Hyperconcentration area (mm^2^)**						
Upper cheek	2037.77 ± 263.73	1931.62 ± 289.1	1908.62 ± 328.31	2236.62 ± 107.78	1954.85 ± 233.88	1894.08 ± 314.18
*p*-value ^1^		**<0.001**	**<0.001**		**<0.001**	**<0.001**
*p*-value ^2^		0.999			0.332	
Lower cheek	2132.08 ± 357.29	2004.27 ± 349.82	1909.35 ± 444.14	2152.42 ± 321.45	2041.62 ± 298.89	1902.31 ± 499.15
*p*-value ^1^		**0.002**	**<0.001**		**<0.001**	**<0.001**
*p*-value ^2^		0.995			0.497	
**Hemi-MASI**	8.93 ± 3.64	6.71 ± 3.03	4.98 ± 2.47	8.93 ± 3.47	6.43 ± 2.98	5.02 ± 2.4
*p*-value ^1^		**0.001**	**<0.001**		**<0.001**	**<0.001**
*p*-value ^2^		**0.005**			**0.021**	
**Wrinkle indentation**						
Crow’s feet	12.53 ± 2.89	11.27 ± 2.47	10.88 ± 2.24	11.82 ± 2.14	10.7 ± 1.73	10.47 ± 1.75
*p*-value ^1^		**0.007**	**<0.001**		**<0.001**	**<0.001**
*p*-value ^2^		0.08			0.183	
Nasolabial fold	43.47 ± 24.23	39.97 ± 21.78	38.75 ± 19.38	43.03 ± 17.95	38.35 ± 14.64	38.23 ± 15.04
*p*-value ^1^		0.055	**<0.001**		**0.003**	**0.005**
*p*-value ^2^		0.157			0.994	
**Wrinkle maximum depth (mm)**						
Crow’s feet	0.18 ± 0.05	0.17 ± 0.06	0.15 ± 0.05	0.18 ± 0.05	0.15 ± 0.05	0.15 ± 0.05
*p*-value ^1^		0.178	**0.009**		**0.017**	**0.002**
*p*-value ^2^		0.173			0.999	
Nasolabial fold	0.25 ± 0.12	0.22 ± 0.09	0.23 ± 0.09	0.24 ± 0.09	0.22 ± 0.09	0.22 ± 0.09
*p*-value ^1^		0.184	0.999		0.716	0.249
*p*-value ^2^		0.636			0.999	
**Pore (mm^3^)**	4.15 ± 2.61	3.8 ± 2.31	3.25 ± 1.87	3.75 ± 1.84	3.16 ± 1.43	2.61 ± 1.35
*p*-value ^1^		0.999	**0.002**		**0.003**	**<0.001**
*p*-value ^2^		**0.025**			0.184	
**Skin texture (Ra)**	9.3 ± 2.15	8.96 ± 1.82	8.58 ± 1.63	9.26 ± 1.59	8.95 ± 1.56	8.29 ± 1.45
*p*-value ^1^		0.237	**0.007**		**0.007**	**<0.001**
*p*-value ^2^		**0.029**			0.214	
**Hemoglobin**						
Concentration	1.15 ± 0.13	1.14 ± 0.13	1.15 ± 0.14	1.15 ± 0.14	1.13 ± 0.12	1.15 ± 0.13
*p*-value ^1^		0.802	0.999		0.999	0.999
*p*-value ^2^		0.995			0.497	
Variation	0.16 ± 0.03	0.15 ± 0.02	0.15 ± 0.03	0.15 ± 0.03	0.14 ± 0.02	0.15 ± 0.03
*p*-value ^1^		0.636	0.113		0.497	0.999
*p*-value ^2^		0.999			0.157	

Data are presented as mean ± SD. ^1^ comparison with baseline; ^2^ comparison between 4 weeks and 16 weeks; all comparisons used one-way repeated measures ANOVA or the Friedman test, depending on normality. *p* < 0.05 are in bold.

**Table 2:** Correlation between the depigmentation effect of MRF and age.

	Thermage		10therma	
	β	*p*-Value ^1^	β	*p*-Value ^1^
*4 weeks—baseline change*				
Melanin level	0.097	0.44	−0.006	0.96
Melanin variation	0.73	0.21	0.03	0.95
Hyperconcentration	1.05	0.04	−0.17	0.69
Hyperconcentration area	0.09	0.785	−0.24	0.3
*16 weeks—baseline change*				
Melanin level	−0.007	0.97	−0.017	0.91
Melanin variation	−0.38	0.58	−0.16	0.8
Hyperconcentration	0.22	0.67	−0.3	0.63
Hyperconcentration area	−0.34	0.59	−0.13	0.83

^1^ linear regression analysis with adjustment for baseline values.

## Data Availability

The raw data supporting the conclusions of this article will be made available by the authors on request.

## Supplementary Materials

The following supporting information can be downloaded at: https://www.mdpi.com/article/10.3390/ijms27020761/s1.

## Abbreviations

The following abbreviations are used in this manuscript:

UV	absorbing ultraviolet
PIH	post-inflammatory hyperpigmentation
RF	radiofrequency
MRF	monopolar radiofrequency
MASI	Melasma Area and Severity Index
hemi-MASI	hemi–Melasma Area and Severity Index
GAIS	Global Aesthetic Improvement Scale
DMEM	Dulbecco’s Modified Eagle Medium
FFPE	formalin-fixed paraffin-embedded
H&E	Hematoxylin and eosin
FM	Fontana–Masson
IHC	Immunohistochemical
TYR	tyrosinase
MMP	matrix metalloproteinase
COL I	collagen type I
COL IV	collagen type IV
GAPDH	glyceraldehyde-3-phosphate dehydrogenase
RT-qPCR	Quantitative reverse transcription PCR
SD	standard deviation
HSP	Heat Shock Protein
αMSH	Alpha-Melanocyte-Stimulating Hormone
MC1R	Melanocortin 1 Receptor
MITF	Microphthalmia-Associated Transcription Factor
