# Supplementary material for: Monopolar Radiofrequency for Facial Hyperpigmentation Treatment: An Integrated Retrospective Clinical Trial and Ex Vivo Study
Source: Int J Mol Sci. 2026 Jan 12;27(2):761. doi: 10.3390/ijms27020761 (PMC12841156; doi:10.3390/ijms27020761)
Supplement: Supplementary file 1 [file ijms-27-00761-s001.zip › ijms-4049974-supplementary.pdf]

Supplementary tables

Table S1. Hemi Melasma Area and Severity Index (Hemi-MASI)

Point	Area of involvement (A)	Darkness (D)	Homogeniety (H)
0	No involvement	Absent	Normal skin color without evidence of hyperpigmentation
1	< 10%	Slight	Specks of involvement
2	10~29%	Mild	Small patchy areas of involvement <1.5 cm diameter
3	30~49%	Marked	Patches of involvement >2 cm diameter
4	50~69%	Severe	Uniform skin involvement without any clear areas
5	70~89%		
6	90~100%%		

Position	Scoring (each side)	Hemi-MASI (each side)
Forehead	0.15 x A x (D + H)	0.15 x A x (D + H) + 0.3 x A x (D + H) + 0.05 x A x (D + H)
Malar	0.3 x A x (D + H)	
Chin	0.05 x A x (D + H)	

Table S2. Global Aesthetic Improvement Scale

Score	Description
1	Very much improved
2	Much improved
3	Improved
4	No change
5	Worse

Table S3. Summary of study procedures and assessment schedule

Procedure	Baseline (treatment)	Follow-up	
Visit	V0	V1	V2
Schedule	Week 0	Week 4	Week 16
1. Written consent	●		
2. Hemi Melasma Area and Severity Index (Hemi-MASI)	●	●	●
3. Investigator Global Aesthetic Improvement Scale (GAIS)		●	●
4. Subject GAIS		●	●
5. Antera 3D®:<br>- Melanin (level, variation, hyperconcentration & hyperconcentration area)<br>- Wrinkles, pore volume, hemoglobin, texture	●	●	●
6. Identification of adverse events	●	●	●

Table S4. Donor demographics

Donor #	Age (years)	Sex	Ethnicity	Tissue source	Biopsy site	Health status
1	49	Female	Korean	TRAM flap	Lower abdomen	No skin disease
2	50	Female	Korean	TRAM flap	Lower abdomen	No skin disease
3	42	Female	Korean	TRAM flap	Lower abdomen	No skin disease

Table S5. Antibodies used in Western blot

Antibody	Company	Catalog no.	Dilution rate
Primary antibodies			
MMP1	Cell signaling	54376	1:1000
COLLAGEN I	Abcam	ab260043	1:1000
Cleaved collagen I C-propeptide	Abcam	ab260043	1:1000
COLLAGEN IV	Abcam	ab6586	1:1000
GAPDH	Cell signaling	2118	1:1000
Secondary antibodies			
Anti-rabbit IgG, HRP-linked	Abcam	7074	1:1000

Table S6. Primers used for RT-qPCR analysis

Primer name		Sequences
HSF	F	TGAAAAGTGCCTCAGCGTAGCC
	R	TGCTCAGCATGGTCTGCAGGTT
HSP47	F	AACCGTGGCTTCATGGTGACTC
	R	TGATGAGGCTGGAGAGCTTGTG
HSP70	F	ATGGTACCGGGAACCGGCATGGCCAAAGCC
	R	ATCCCGGGAATGGTGGGGCCTGACC
MC1R	F	ATCCTTCCTGGACAGGACT
	R	CATTTAGTCCATCCTCTTTG
α-MSH	F	TACGTCATGGGCCACTTC
	R	AGTGCTCCATCCTGTAGG
MITF	F	GACGGTCACTGCAGACTTTG
	R	GCCATGACCAGGATGAC
MMP2	F	AGCGAGTGGATGCCGCCTTTAA
	R	CATTCCAGGCATCTGCGATGAG
MMP9	F	GCCACTACTGTGCCTTTGAGTC
	R	CCCTCAGAGAATCGCCAGTACT
P53	F	CCTCAGCATCTTATCCGAGTGG
	R	TGGATGGTGGTACAGTCAGAGC
P16	F	CTCGTGCTGATGCTACTGAGGA
	R	GGTCGGCGCAGTTGGGCTCC
P21	F	AGGTGGACCTGGAGACTCTCAG
	R	TCCTCTTGGAGAAGATCAGCCG
GAPDH	F	GTCTCCTCTGACTTCAACAGAG
	R	ACCACCCTGTTGCTGTAGCCAA

Abbreviations: F, forward; R, reverse

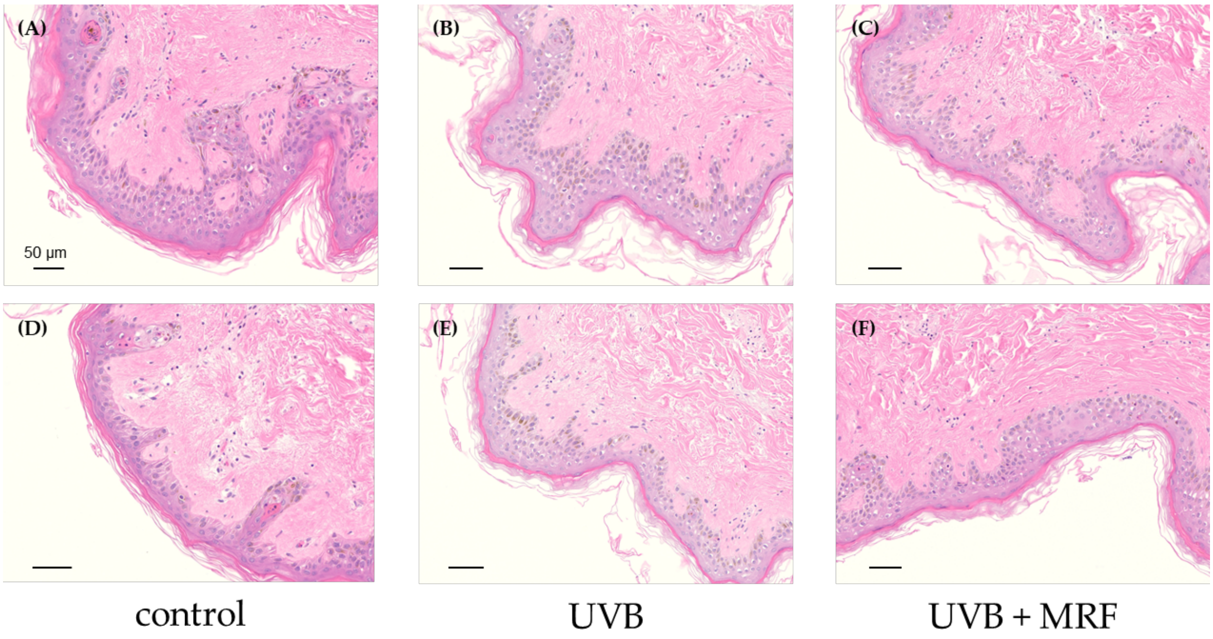

Figure S1. Histological analysis of skin tissues by H&E staining

Representative H&E-stained images of ex vivo human skin tissues from two independent biological replicates. Panels (A–C) and (D–F) correspond to skin samples from Donor #1 and Donor #2, respectively. Panels (A, D) show control skin, panels (B, E) show UVB-irradiated skin, and panels (C, F) show UVB + MRF-treated skin. UVB irradiation was associated with epidermal thickening and structural alteration, whereas UVB + MRF-treated samples showed relatively preserved epidermal and dermal morphology compared with UVB-only samples.

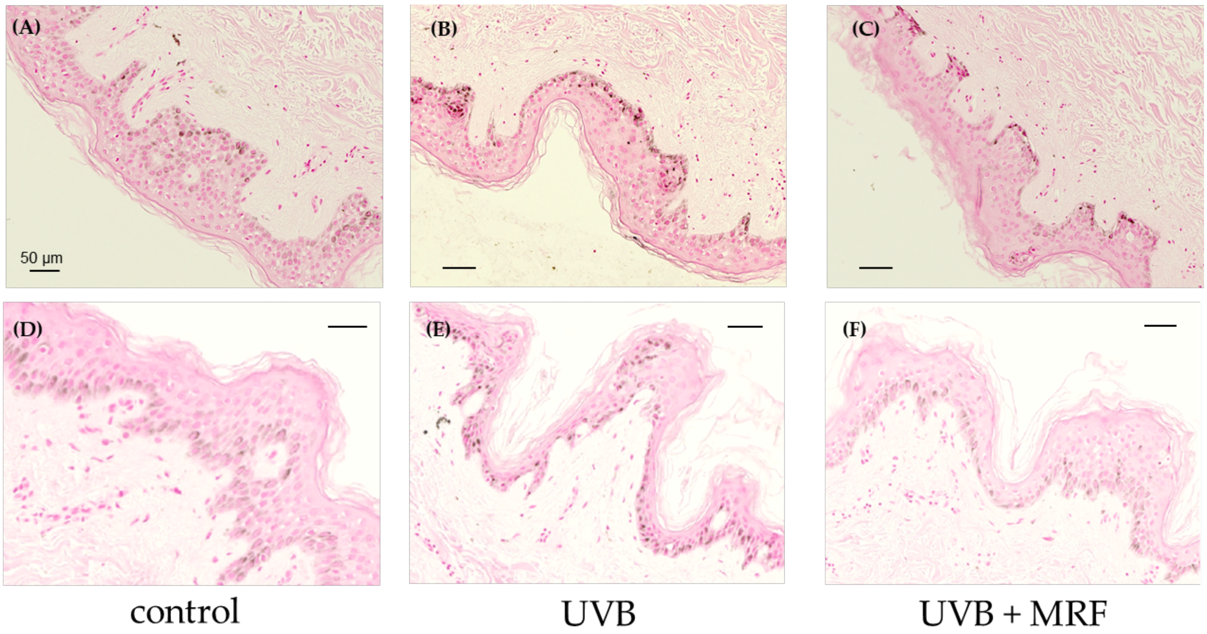

Figure S2. Melanin visualization by Fontana–Masson staining

Representative FM-stained images of ex vivo human skin tissues from independent biological replicates. Panels (A–C) and (D–F) correspond to skin samples from Donor #1 and Donor #2, respectively, each showing control, UVB-irradiated, and UVB + MRF-treated groups. UVB irradiation was associated with increased melanin deposition, whereas UVB + MRF-treated samples exhibited reduced FM staining intensity compared with UVB-only samples.

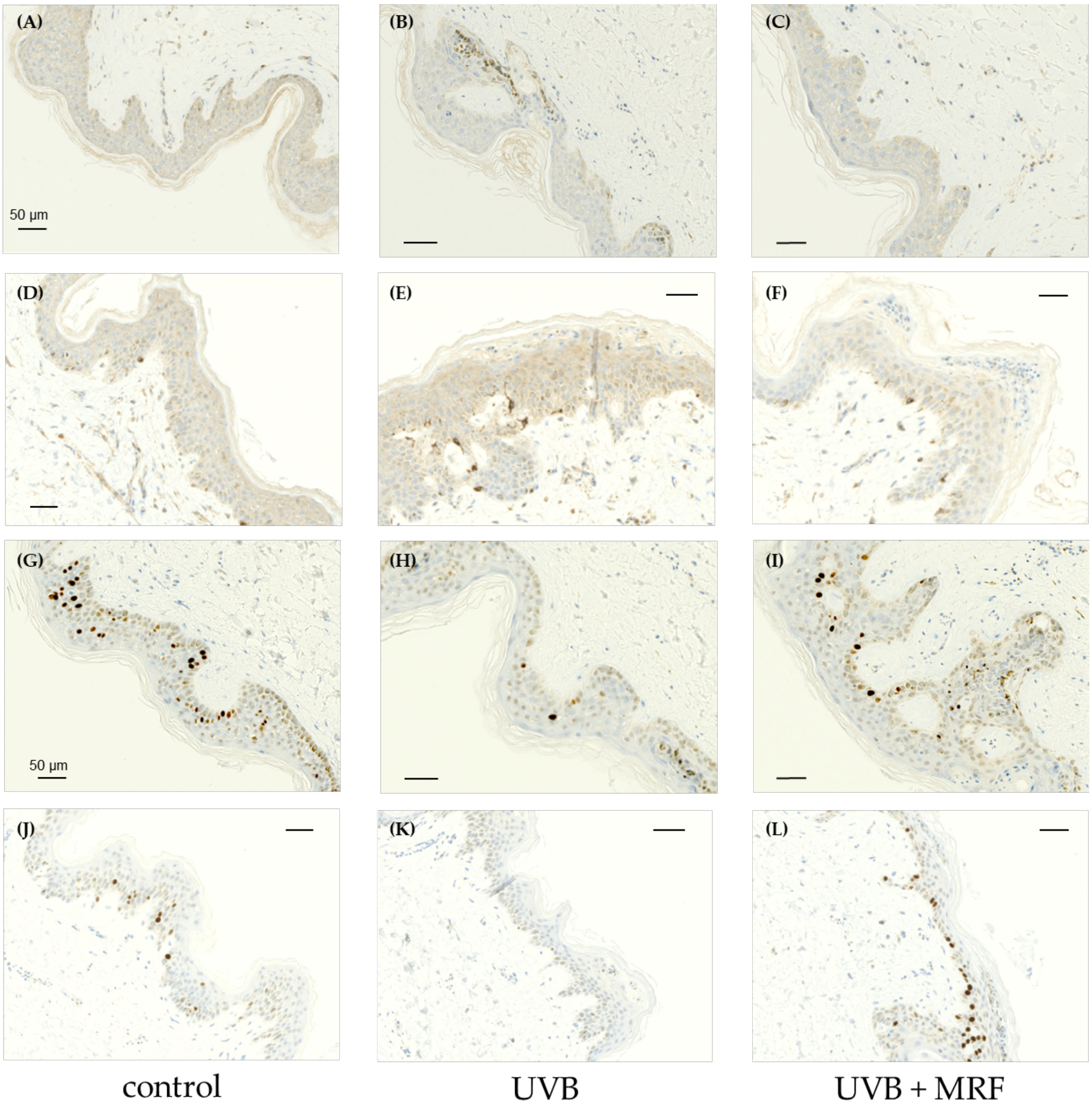

Figure S3. Immunohistochemical staining of TYR and Ki-67 in skin tissues

Representative IHC images of TYR (A-F) and Ki-67 (G-L) in control (left panel), UVB-irradiated (middle panel), and MRF-treated groups (right panel) using ex vivo human skin tissues from two independent biological replicates (Donor #1 and Donor #2). TYR expression increased after UVB irradiation and decreased following UVB + MRF treatment. Ki-67–positive cells decreased after UVB exposure and increased in UVB + MRF-treated skin, indicating enhanced proliferative activity. TYR, tyrosinase.

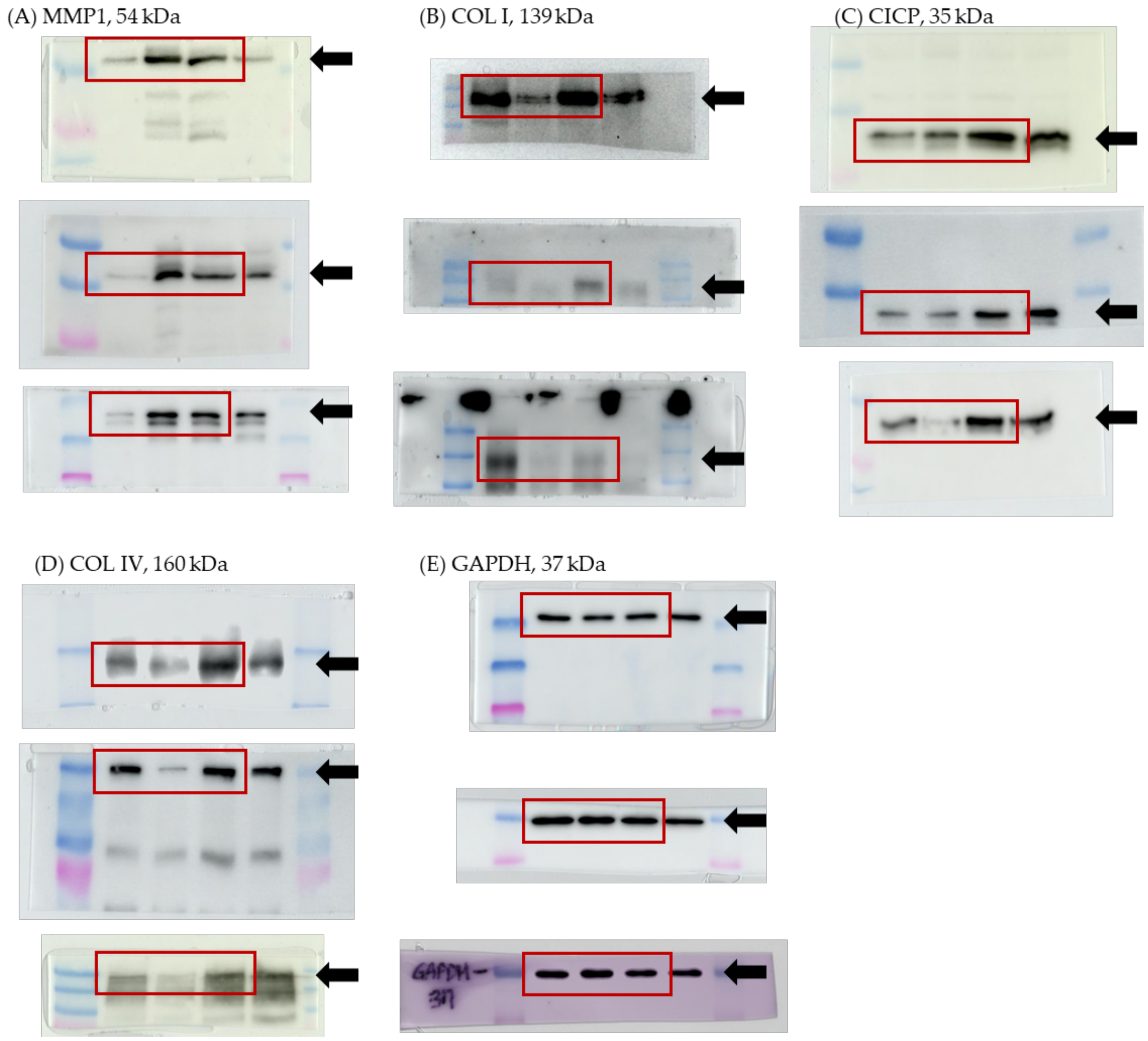

Figure S4. Raw Western blot images corresponding to the data presented in Figure 12 Western blot images for (A) MMP-1, (B) COL I, (C) CICP, (D) COL IV, and (E) GAPDH. The expected molecular weights of each target protein are indicated. Lane 1 represents the control group; Lane 2 represents the UVB-irradiated group; Lane 3 represents the MRF-treated central region; and Lane 4 represents the MRF-treated margin region. Black arrows indicate the specific bands used for quantification, and the red rectangular box highlights the lane presented in the main text. MMP-1 expression increased following UV irradiation and was reduced after MRF treatment. In contrast, the expression levels of COL I, CICP, and COL IV were decreased after UVB exposure but were preserved following MRF treatment, indicating a restorative effect of MRF on extracellular matrix integrity. MMP-1, matrix metalloproteinase-1; COL I, collagen I; CICP, cleaved collagen I C-propeptide; GAPDH, glyceraldehyde 3-phosphate dehydrogenase.
